# Anatomic comparison of veins of Labbé between autopsy, digital subtraction angiography and computed tomographic venography

**DOI:** 10.1186/s12938-017-0374-3

**Published:** 2017-06-26

**Authors:** Qiong Fang, Anhong Jiang, Wei Tao, Lin Xin

**Affiliations:** 1 Department of Anatomy, Anhui Medical College, Hefei, 230601 Anhui China; 2 Department of Radiology, The Second Affiliated Hospital of Anhui Medical University, Hefei, 230601 China; 3 Department of Anatomy, School of Medicine, Anhui University of Science & Technology, 25 Dongshan Road, Huainan, 232001 China; 4 Department of Cardiology, Guangzhou General Hospital of Guangzhou Military Region, PLA, Guangzhou, 510010 China

**Keywords:** Veins of Labbé, Supratentorial approach, Microanatomy, Digital subtraction angiography, Computed tomographic venography

## Abstract

**Objective:**

The drainage portion of the vein of Labbé varies, and it is difficult to predict whether the operation is likely to damage this vein. The aim of this study was to correlate the microanatomy of the vein of Labbé with digital subtraction angiography (DSA) and computed tomographic venography (CTV), in order to provide a basis for the preservation of the vein of Labbé during a supratentorial surgical approach.

**Methods:**

A total of 30 human cadavers (60 sides) and 61 living patients (110 sides) were examined in this study. Each cadaver head was injected with blue latex via the superior sagittal sinus and the internal jugular veins. The venograms of each patient were obtained from the venous phases of DSA (60 sides for 36 patients) or CTV (50 sides for 25 patients).

**Results:**

The patients were divided into four subgroups based on the location where a vein entered the dural sinus: the transverse sinus group, the tentorial group, the petrosal group, and the upper-transverse sinus group. The veins of Labbé in transverse sinus group and petrosal group directly entered dural sinus. The veins of Labbé in tentorial group and upper-transverse sinus group indirectly entered transverse sinus via the tentorium sinus or the upper-transverse sinus. These sinuses were meningeal veins running through two layers of the cerebral dura mater. The length of meningeal veins in these groups was 10.0 ± 7.2 mm. The veins of Labbé were mainly localized around the STP junction, which was the confluence of sigmoid sinus, transverse sinus, and superior petrosal sinus. The distance between the dural entrance of veins and the STP junction was 16.8 ± 10.2 mm. There was no significant difference in the results of the DSA and CTV examinations when compared to the observations in cadavers.

**Conclusions:**

Preoperative venograms are useful to design an individualized surgical approach for the preservation of the vein of Labbé. In general, the supratentorial median approach has the least chance to damage this vein. However, when preoperative venograms show that the vein of Labbé is too close to the confluence of sinuses or the meningeal veins are too long, an alternative approach should be chosen.

## Background

Anatomic and histological observation has shown that the cerebral venous wall is thin and the course is flat [1]. During the operation process of a given neurosurgical approach, issues such as the separation of blood vessels, cerebral traction, and expansion of the operation space may necessitate electrocoagulation and ligation of the cerebral veins, resulting in intraoperative bleeding and differing degrees of postoperative complications [2–8]. It has been proven that after damage to the bridging veins, which are the final segment of cerebral veins bridging between brain and dural sinus, venous occlusion and regional blood flow decreased, leading to cerebral edema, cerebral hemorrhage, cerebral venous infarction, and other postoperative complications [9–11]. In addition, cerebral traction and injury to the bridging veins will further aggravate the injury to the vascular endothelial cells and affect postoperative rehabilitation [11].

The vein of Labbé is a type of bridging vein that is first discovered and should be protected during the operation. It was originally defined as one of a group of anastomosis veins that connect the superficial middle cerebral vein around the lateral sulcus and the lateral sinus. It was also called the inferior anastomotic vein [12] which drained the blood from lateral temporal lobe and the gyrus around lateral sulcus. In the clinical supratentorial surgical approaches, the separation of blood vessels, cerebral traction, expansion of the operation space and other risks, may injure the vein of Labbé and result in aphasia, logagraphia, encephaledema, and other complications. Neurosurgeons should protect the vein of Labbé as much as possible [13, 14].

The drainage portion of the vein of Labbé varies [15–17], so it is difficult to predict whether the operation is likely to damage this vein. Preoperative imaging can evaluate the number, diameter, distribution, and drainage of the cerebral veins [18], which play an important role in the design of the operative approach. However, there have been few reports on imaging studies of the vein of Labbé. Therefore, the aim of this study was to correlate the microanatomy of the vein of Labbé with venograms as obtained by digital subtraction angiography (DSA) and computed tomographic venography (CTV), in order to provide a basis for the preservation of the vein of Labbé during supratentorial surgical approaches.

## Methods

### Microanatomy

Thirty cadavers fixed with formalin were provided by the Department of Anatomy of Anhui Medical College. There were 23 male cadavers and 7 female. The age range was 40 ± 11 years old (16–59 years).

After removing the cranium, cavity congestion in the superior sagittal sinus and internal jugular veins was flushed by intubation, and blue latex was perfused through these vessels. The dura mater near the temporal occipital lobe was carefully separated from the skull after 48 h, in order to protect the veins below them. The skull above the transverse sinus was removed along both sides of the mastoid. The dura mater was cut 25 mm above the transverse sinus.

**Fig. 1:**
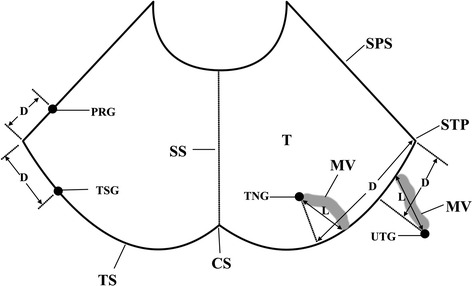
Sketch map of dural entrance of veins of Labbé. The part behind the transverse sinus shows the dura mater above the transverse sinus. The dural entrances (*filled circle*) were in the surface of transverse sinus (TSG, transverse sinus group), superior petrosal sinus (PRG, petrosal group), tentorium cerebelli (TNG, tentorial group) or dura mater above the transverse sinus (UTG, upper-transverse sinus group). *CS* confluence of sinuses, *D* distance from dural entrance to STP, *L* length of meningeal vein, *MV* meningeal vein, *SPS* superior petrosal sinus, *SS* straight sinus, *STP* confluence of sigmoid sinus, transverse sinus and superior petrosal sinus, *T* tentorium cerebelli, *TS* transverse sinus

According to the Rhoton standard [19], the maximal anastomotic branch between the superficial middle cerebral vein and the transverse sinus is defined as the vein of Labbé. The pattern by which the vein of Labbé entered dural sinus were observed, the location of the dural entrance was noted, and the diameter was measured at the site 5 mm before the dural entrance. When the vein of Labbé indirectly entered transverse sinus through the veins in dura mater, the length of this veins was measured (Fig. 1). If the dural entrance was in the dural sinus, the shortest distance from the dural entrance to the confluence of sigmoid sinus, transverse sinus, and superior petrosal sinus (STP junction) was measured. If the dural entrance was not in the dural sinus, the shortest distance from the vertical projection of the dural entrance on the transverse sinus to the STP junction was measured (Fig. 1).

### Digital subtraction angiography

DSA views of veins from 60 sides with no cerebral venous diseases were selected from 36 patients. There were 22 male patients and 14 female. The age range was 45 ± 16 years old (11–76 years). The indications for examination were aneurysm (8 patients), lacunar cerebral infarction (21 patients), and only suspicion of an intracranial lesion (7 patients). In 12 patients, only unilateral vein was obtained. In other 24 patients, bilateral veins were obtained.

A 500 MA digital subtraction X machine and a Siemens Axiom Artis DSA machine were used. In addition, selective cerebral angiography was performed by puncturing the femoral arteries using the Seldinger technique. The contrast medium was Omnipaque from GE Healthcare AS, and the imaging rate was three frames per second. Views were taken of the bilateral internal carotid artery and from the venous phase of a series of anteroposterior and lateral radiography. The flow rates of the internal carotid artery and vertebral artery angiography were 3–5 and 2–4 ml/s, respectively. The total amounts of the contrast medium were 7–l0 and 5–7 ml, respectively.

### Computed tomographic venography

CTV images of 25 cases (50 sides) with no cerebral venous diseases were observed. There were 14 male patients and 11 female. The age range was 45 ± 17 years old (14–74 years). The indications for examination were aneurysm (5 patients), lacunar cerebral infarction (12 patients), and only suspicion of an intracranial lesion (4 patients).

Spiral CT scanning was performed with 64-slice GE-light speed CTV. The scanning parameters were as follows: screw pitch was 0.531, layer thickness was 2.5 mm, tube voltage was 120 kv, tube current was 335 mA, intravenously injected Omnipaque was 100 ml (350 mg I/ml), flow velocity was 4 ml/s, and the delay time was 50 s. The scanning range was from the inferior border of the mandible to the calvarium. After the scanning was completed and the images were reconstructed into 0.625 mm, the data were transferred to an AW 4.2 workstation, and 3D images of the cerebral veins were reconstructed with volume rendering.

### Statistical treatments

The statistical analysis was performed with SPSS 19.0 statistical software. The numerical values of both the left and right sides were obtained with a t test. The observed microanatomy and the results of DSA and CTV were analyzed with a one-way analysis of variance or Chi square test. The statistical results were shown in $\bar{x}_{{{ \pm\text{s}}} \left( {\text{min} -\text{max} }\right)}$.

## Results

### The groupings, numbers, and diameters of the veins of Labbé

**Fig. 2:**
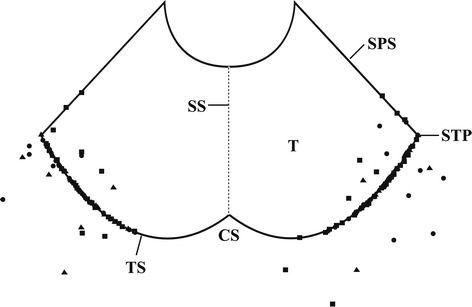
Scatter plots of the distribution of veins of Labbé. The part behind the transverse sinus shows the dura mater above the transverse sinus. *Filled circle* Cadaver, *filled square* DSA, *filled triangle* CTV, *CS* confluence of sinuses, *SPS* superior petrosal sinus, *SS* straight sinus, *STP* confluence of sigmoid sinus, transverse sinus and superior petrosal sinus, *T* tentorium cerebelli, *TS* transverse sinus

**Fig. 3:**
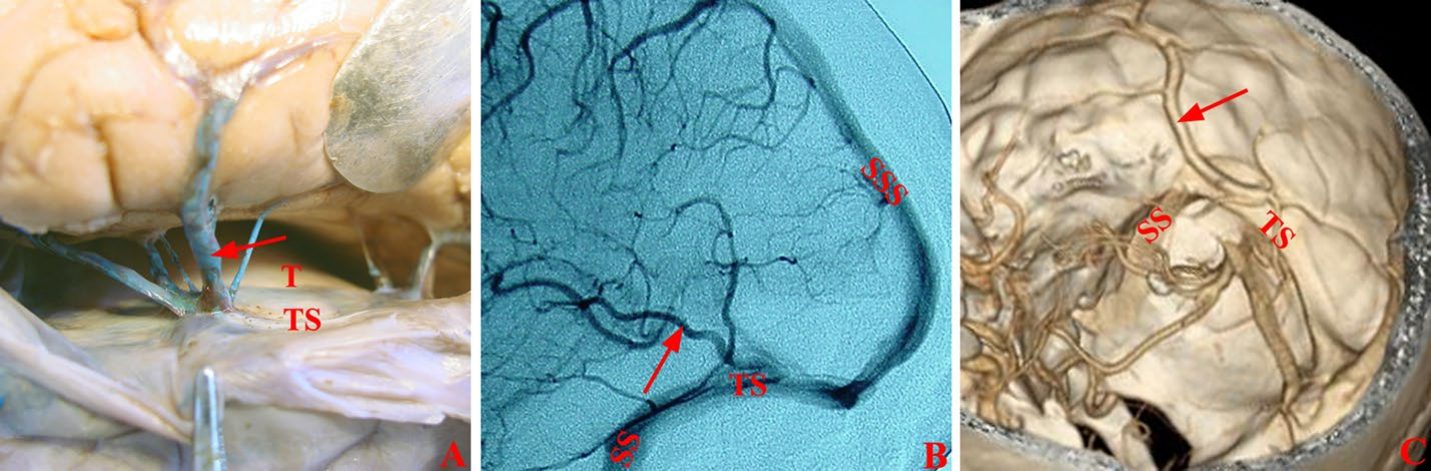
Vein of Labbé in transverse sinus group. The vein of Labbé (*arrow*) directly entered transverse sinus in Cadaver (**A**), DSA (**B**), and CTV (**C**). *SS* sigmoid sinus, *SSS* superior sagittal sinus, *T* tentorium cerebelli, *TS* transverse sinus

**Fig. 4:**
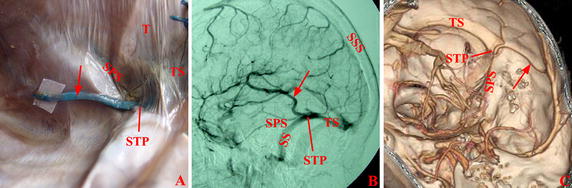
Vein of Labbé in petrosal group. The veins of Labbé (*arrow*) directly entered the superior petrosal sinus in Cadaver (**A**), DSA (**B**), and CTV (**C**). *SPS* superior petrosal sinus, *STP* confluence of sigmoid sinus, transverse sinus and superior petrosal sinus, *SS* sigmoid sinus, *SSS* superior sagittal sinus, *T* tentorium cerebelli, *TS* transverse sinus

**Fig. 5:**
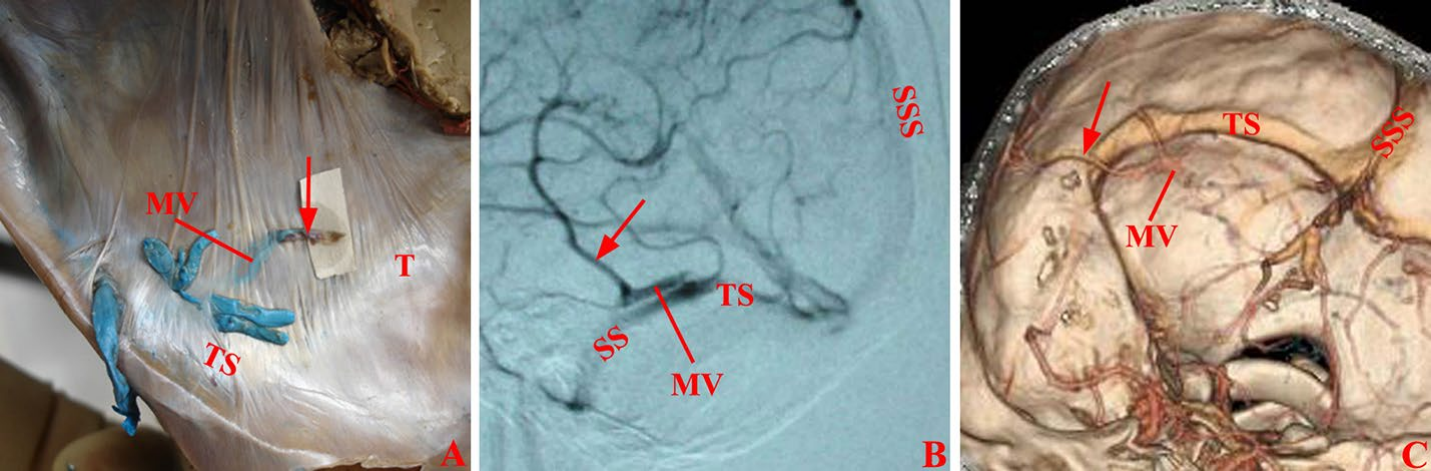
Vein of Labbé in tentorial group. The veins of Labbé (*arrow*) indirectly entered transverse sinus through the meningeal veins on the tentorium cerebelli in Cadaver (**A**), DSA (**B**), and CTV (**C**). *MV* meningeal vein, *SS* sigmoid sinus, *SSS* superior sagittal sinus, *T* tentorium cerebelli, *TS* transverse sinus

**Fig. 6:**
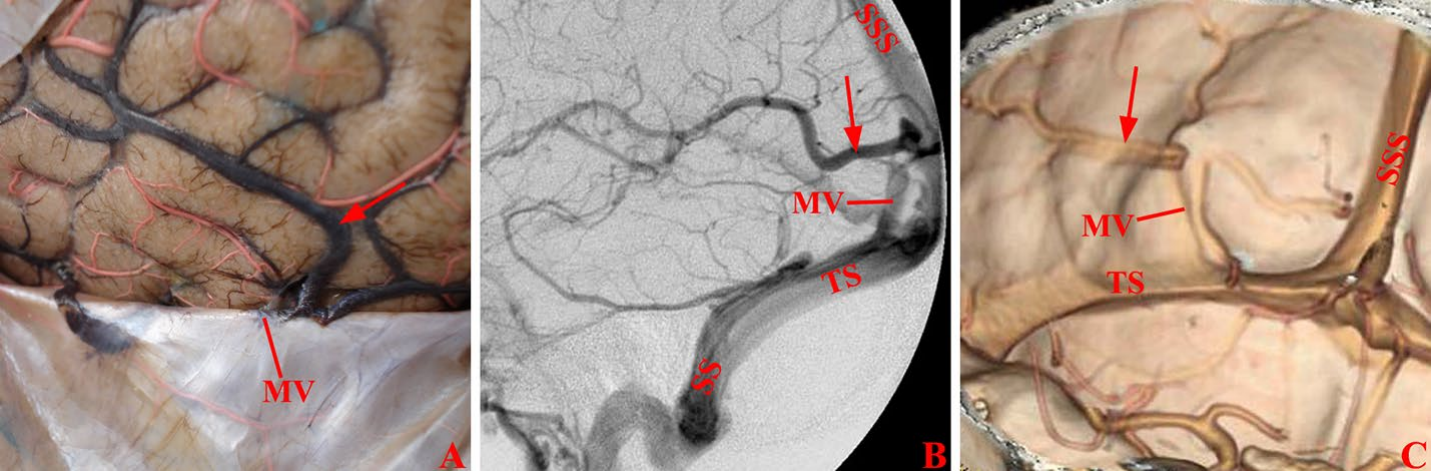
Vein of Labbé in upper-transverse sinus group. The veins of Labbé (*arrow*) indirectly entered transverse sinus through the meningeal veins on the dural mater above transverse sinus in Cadaver (**A**), DSA (**B**), and CTV (**C**). *MV* meningeal vein, *SS* sigmoid sinus, *SSS* superior sagittal sinus, *T* tentorium cerebelli, *TS* transverse sinus

The patients were divided into four subgroups based on the pattern and the location of the entrance of a vein into dura mater (Fig. 2). The direct entry group comprised patients for whom the veins of Labbé directly entered transverse sinus (transverse sinus group, Fig. 3) or superior petrosal sinus (petrosal group, Fig. 4). The entrance was located in the dura mater where the transverse sinus or superior petrosal sinus formed. For the indirect entry group, the veins of Labbé indirectly entered transverse sinus or superior petrosal sinus. The entrance was located in the tentorium cerebelli (tentorial group, Fig. 5) or the dura mater above the transverse sinus (upper-transverse sinus group, Fig. 6).

In DSA and CTV images, the superficial middle cerebral veins intersected with the transverse sinus through the veins of Labbé (Figs. 3, 4, 5, 6), except for no vein of Labbé was present in one case. In this patient, the left inferior cerebral vein entering transverse sinus was very small and there was no anastomotic branch between transverse sinus and superficial middle cerebral veins. There was no significant difference in the number and diameter of the veins of Labbé as measured by CTV and DSA (Fig. 2; Table 1), when compared with that of the cadavers.

**Table 1:** Number and diameter of the veins of Labbé

Groups	Cadavers (n = 60)	DSA (n = 60)	CTV (n = 50)
Direct	2.7 ± 0.7 (1.5–4.4) [48]	2.9 ± 0.5 (1.7–4.2) [50]	2.8 ± 0.4 (1.9–4.2) [41]
TSG	2.7 ± 0.7 (1.5–4.4) [46]	2.9 ± 0.5 (1.7–4.2) [45]	2.8 ± 0.5 (1.9–4.4) [39]
PRG	2.7 ± 0.4 (2.5–3.0) [2]	2.8 ± 0.5 (2.4–3.7) [5]	3.1 ± 0.0 (3.1–3.1) [2]
Indirect	3.3 ± 0.8 (2.2–4.6) [12]	2.7 ± 0.5 (2.0–3.5) [10]	2.6 ± 0.4 (2.0–3.1) [8]
TNG	3.0 ± 0.8 (2.2–3.8) [3]	2.7 ± 0.3 (2.3–3.1) [6]	2.4 ± 0.6 (2.0–2.8) [2]
UTG	3.4 ± 0.8 (2.4–4.6) [9]	2.6 ± 0.8 (2.0–3.5) [4]	2.7 ± 0.3 (2.2–3.1) [6]
Total	2.8 ± 0.7 (1.5–4.6) [60]	2.8 ± 0.5 (1.7–4.2) [60]	2.8 ± 0.4 (1.9–4.4) [49]

In the bracket was the number of veins of Labbé in each group. No difference in comparison with the number of cadavers, DSA and CTV (Chi square test, *p* > 0.05). No difference in comparison with the diameter of cadavers, DSA and CTV of (ANOVA, *p* > 0.05); No difference was found between two sides (*t* test, *p* > 0.05)

*TSG* transverse sinus group, *PRG* petrosal group, *TNG* tentorial group, *UTG* upper-transverse sinus group

### Morphology of meningeal veins

In microscopic anatomy, 20% of the veins of Labbé indirectly entered transverse veins through the meningeal veins. The dural veins went between the dural layers, with a flat shape, and their diameter was larger than that of the veins of Labbé. The average length was 10.0 ± 7.2 mm, with a range up to 23.6 mm (Table 2). This was called the tentorial sinus when it was located in the tentorium, while it was called the upper-transverse sinus when it was located in the dura mater above the transverse sinus.

**Table 2:** Length of the meningeal veins

Meningeal veins	Cadavers (n = 60)	DSA (n = 60)	CTV (n = 50)
Tentorium sinus	7.5 ± 3.2 (5.2–11.1)	6.1 ± 2.4 (3.8–10.2)	5.6 ± 2.0 (4.2–7.0)
Upper-transverse sinus	10.9 ± 8.1 (1.7–23.6)	10.3 ± 6.1 (5.4–19.3)	10.4 ± 6.5 (4.4–21.8)
Total	10.0 ± 7.2 (1.7–23.6)	7.8 ± 4.5 (3.8–19.3)	9.2 ± 5.9 (4.2–21.8)

No difference in comparison with the cadavers, DSA and CTV (ANOVA, *p* > 0.05); No difference was found between two sides (*t* test, *p* > 0.05)

In the DSA and CTV images, there was a flat part with a large diameter before the entrance where the veins of Labbé entered the transverse sinus in 17 and 16% of patients, respectively. The tentorium sinus was located in front of the transverse sinus, and entered the transverse sinus backward (Fig. 5). The upper-transverse sinus was located above the transverse sinus, and entered transverse sinus downward (Fig. 6). There was no statistical difference when comparing the length of meningeal veins obtained by DSA and CTV with observations from cadavers (Table 2).

### Distance between the veins of Labbé and the STP junction

The dural entrance of veins of Labbé were distributed near the STP junction, and there were no entrance around the confluence of sinuses (Fig. 2). The average distance between the veins of Labbé and the STP junction was 16.8 mm, and the distance exceeded 35 mm in 6% of cases. There was no statistical difference when comparing the combined results obtained by DSA and CTV with measurements from cadavers (Table 3).

**Table 3:** Distance between the veins of Labbé and STP junction

Groups	Cadavers (n = 60)	DSA (n = 60)	CTV (n = 50)
Direct	18.7 ± 10.0 (0–40.1)	19.2 ± 11.0 (1.4–42.6)	15.4 ± 8.4 (0–3.4)
TSG	19.4 ± 9.6 (1.8–40.1)	19.9 ± 11.3 (1.4–42.6)	16.2 ± 7.8 (3.6–34.0)
PRG	2.0 ± 2.8 (0–4.0)	12.5 ± 5.6 (6.4–18.8)*	0^a^
Indirect	9.4 ± 7.6 (0.7–25.5)	22.8 ± 13.8 (1.4–46.9)*	18.7 ± 11.2 (1.4–33.1)
TNG	9.9 ± 0.6 (9.2–10.4)	14.0 ± 8.1 (1.4–26.3)	23.9 ± 6.1 (19.6–28.2)
UTG	9.2 ± 8.9 (0.7–25.5)	36.0 ± 9.0 (26.7–46.9)*	17.0 ± 12.4 (1.4–33.1)
Total	16.8 ± 10.2 (0–40.1)	19.7 ± 11.4 (1.4–46.9)	16.0 ± 8.8 (0–34.0)

* Compared with cadavers, *p* < 0.05; No difference was found between two sides (*t* test, *p* > 0.05)

^a^Two veins drained into the STP junction (confluence of sigmoid sinus, transverse sinus and superior petrosal sinus)

However, in some special groups, there was a significant difference between the distance obtained by DSA and that of cadavers. When the veins of Labbé indirectly entered, the average distance observed in cadavers was 9.4 mm, and the DSA result was 22.8 mm. In the veins of Labbé of the petrosal group, the average distance in cadavers was 2.0 mm, and the DSA result was 12.5 mm. In the veins of Labbé in the upper-transverse sinus group, the average distance in cadavers was 9.2 mm, and the DSA result was 36.0 mm. Both of these difference were statistically significant (Table 3).

## Discussion

### Design of supratentorial surgical approaches

With the rapid development of medical imaging, clinicians have gradually directed their attention to the cerebral veins. In previous surgeries, methods that sacrificed the cerebral veins were used to gain ideal surgical fields. This caused venous infarction, brain edema, cerebral hemorrhage, and other complications. Recovery after surgery was seriously affected, and death even resulted due to these complications [6, 13, 14]. Therefore, neurosurgeons have begun to protect the cerebral veins during operations. The commonly used methods are as follows [20]. Free bridging veins: during the operation, the veins bridging the arachnoid mater and the dura mater are dissociated in order to increase the activity degree and enlarge the surgical field to a certain extent, in the case of no damage to the bridging veins. Avoid large bridging veins: the larger-diameter veins have a wider drainage area, leading to serious complications after injury, so the larger-diameter veins are avoided in the surgical approach.

The vein of Labbé is the main drainage vein of the temporal lobe, and the diameter is relatively large. Complications such as aphasia, agraphia, and brain edema appear after an injury to this vein [13, 14]. Most neurosurgeons believe that to avoid complications and promote postoperative recovery, the vein of Labbé should be protected as much as possible [21]. Drainage of the vein of Labbé varies, and it is difficult to predict whether the operation is likely to damage the vein. Preoperative imaging can evaluate the number, diameter, distribution, and drainage of the cerebral veins [22]. These are the key factors that affect the design of operative approaches. This paper found that most of the veins were distributed near the STP junction, and the average distance from the vein to STP junction was less than 20 mm. There was no vein of Labbé around the confluence of sinuses. Therefore, in an operation on the cerebellum, the median approach has the lowest chance of injuring the vein of Labbé. During the operation, after cutting the dura mater covering the occipital lobe, the vein of Labbé is dissociated, to raise the occipital lobe to a certain extent. This also permits the operative field to be expanded without injuring the vein.

However, the median approach should not be used in two specific situations. First, the median approach should be avoided if the dural entrance of the vein of Labbé is far from the STP junction and close to the confluence of sinuses. This situation can be identified when the distance between the vein of Labbé and the STP junction is more than 35 mm. This case is relatively rare, and it accounts for only 6% of cases. Nevertheless, the vein of Labbé will be easily injured in these cases, because it is too close to the median operative approach. Secondly, the median approach is contraindicated if the vein of Labbé indirectly entered the transverse sinus through the meningeal veins and has a greater length. The dural veins go between the dural layers, so it is difficult to separate these from the dura mater. The average length of the dural veins is 10 mm, while the longest is 23.6 mm. The appearance of meningeal veins reduces the length of the free segments of the vein of Labbé, and limits the raise of occipital lobe, then the operative field is greatly reduced. When either of these two issues is observed in radiographic images, a change in approach is recommended, such as by relying on the inferior cerebellar approach or the posterior approach through the corpus callosum.

### Clinical significance of DSA and CTV for the veins of Labbé

Reports differ on the sensitivity of imaging for the varying positions of cerebral veins in imaging observation. Using the microsurgical anatomy as a standard, the sensitivity of the DSA for observation of the Galen venous system is only about 40% [22]. However, the sensitivity of DSA for the bridging veins draining into the superior sagittal sinuses reaches 80% [23], which may be related to the diameters or the different positions of cerebral veins. In this study, the superficial middle cerebral veins intersecting with the transverse sinus through the veins of Labbé were observed in DSA and CTV images, except in one case where the left inferior cerebral vein entering transverse sinus was very small and there was no vein of Labbé present. The sensitivity observed in this study was nearly 100%. DSA could distinguish those blood vessels with diameters of more than 0.2 mm. The 64-slice spiral CT could distinguish blood vessels with diameters between 0.3 and 0.5 mm [24]. The average diameter of the veins of Labbé was 2.8 mm, which is greater than the spatial resolution of DSA and CTV. As a result, these veins can be clearly observed in DSA and CTV images. There was no significant difference in the diameter of the veins of Labbé as measured by CTV and DSA, when compared to direct observation in cadavers. This further demonstrates that the most important factor that affects the observation of vascular images is the diameter [25].

This study found that it is difficult to distinguish the four groups in the DSA and CTV images. In these images, there was a flat part with a large diameter before entering transverse sinus, called the meningeal veins. As the dura mater cannot be displayed in these forms of imaging, the tentorium sinus and the upper-transverse sinus are distinguished through the location of meningeal veins and the entry direction to the transverse sinus. In CTV, the location of the meningeal veins is identified accurately through rotation of 3D images, and the tentorium sinus and upper-transverse sinus are next distinguished. However, when the diameter of the meningeal veins is small, it is difficult to distinguish the meningeal veins due to the interference of high-density bone.

The spatial resolution of DSA is higher than that of CTV, and there is no interference of bone. The sensitivity for meningeal veins is higher, but there are two problems. First, the tentorium is high in some cases, and the dural veins in the images travel downward. It is easy to mistake the tentorium sinus for the dural sinus. Secondly, when the upper-transverse sinus is short, it is difficult to accurately identify whether it travels downward or backward in radiographic images. It is easy to mistake the upper-transverse sinus for the tentorium sinus. Based on the pattern that a vein enters dural sinus, the veins of Labbé were divided into two groups: the direct entry group and the indirect entry group. There was no significant difference in the number of the veins of Labbé and the length of the meningeal veins as measured by CTV and DSA in comparison with results from cadavers. Therefore, this study suggests that in the preoperative imaging examinations before supratentorial surgical approaches, there is no need to divide the patients into four groups. It is only necessary to classify them into two groups according to the way in which the vein of Labbé enters the dura mater, identify the meningeal veins and measure the length of them.

DSA is the gold standard for cerebral vascular imaging, but it is an invasive test. In order to obtain the ideal cerebral venous images, it usually necessary to increase the dosage of the contrast agent and the X-ray irradiation time, which limits the application of DSA in the observation of cerebral veins [25, 26]. By contrast, CTV scanning is very fast, and the price is relatively low. The advent of a new type of 64-slice spiral CT has greatly improved the spatial resolution of the images, and it can display smaller vessels. Magnetic resonance imaging has no radiation effect, and images of blood vessels are developed without a contrast agent. This study shows that the observation effect of CTV on the vein of Labbé is the same as with DSA, and CTV is even better than DSA in measuring the distance between the vein of Labbé and the STP junction. Overall, the distance between the vein of Labbé and the STP junction as measured by DSA and CTV has no significant difference when compared with cadavers. By contrast, the distances of the veins of Labbé measured by DSA in the petrosal group and the upper-transverse sinus group have significant differences when compared with cadavers. However, the veins in the petrosal group are located in the STP junction or superior petrosal sinus. Thus, this difference will not affect supratentorial median surgical approaches. In the veins of Labbé in the upper-transverse sinus group, the length of the meningeal veins is usually greater. The average length is more than 10 mm, indicating that the median approach should not be used whenever possible. Therefore, this difference also didn’t influence the design of supratentorial surgical approaches.

## Conclusions

This study provides an important basis for the design of operative approaches that will ensure the protection of the vein of Labbé, through observation via DSA and CTV of the microanatomy of the vein of Labbé. The results suggest that DSA or CTV examination before the operation is helpful to design the operative approach. For example, in an operation on the cerebellum, the lowest chance of injury to the vein of Labbé is with the median approach. However, when the imaging examination reveals that the vein of Labbé is too close to the confluence of sinuses or the dural veins are too long, the operative approach should be redesigned.

## Abbreviations

DSA: digital subtraction angiography
CTV: computed tomographic venography
STP: confluence of sigmoid sinus, transverse sinus, and superior petrosal sinus
